# Supplementary material for: Mental health in higher education students and non-students: evidence from a nationally representative panel study
Source: Soc Psychiatry Psychiatr Epidemiol. 2021 Feb 15;56(5):879–82. doi: 10.1007/s00127-021-02032-w (PMC8068655; doi:10.1007/s00127-021-02032-w)
Supplement: Supplementary file 1 — Supplementary file1 (PDF 202 KB) [file 127_2021_2032_MOESM1_ESM.pdf]

Supplementary material:

Mental health in higher-education students and non-students: evidence from a nationally representative panel study

Authors: Evangeline Tabor*[1], Praveetha Patalay**[1,2], David Bann**[1]

*Corresponding author. Email: evangeline.tabor.18@ucl.ac.uk

**Contributed equally.

[1]Institute of Social Research, University College London, London, UK

[2]Faculty of Population Health Sciences, University College London, London, UK

Methods

Highest parental educational qualification was derived by sourcing the earliest educational qualification available for each of the respondents' parents across the nine waves. The parent with the highest qualification was selected and used to provide the highest parental educational qualification. Where no parent qualification was available, self-reported parental education was used. Highest parental educational qualification was categorized into the following categories: Degree or equivalent, A levels or equivalent, GCSE or equivalent, No qualification, Other. When deciding the highest qualification, Other is listed where applicable except where a respondent also has a parent who has a degree or equivalent.

Ethnicity is a derived category with eighteen categories. In this analysis ethnicity was recategorized into six categories: White British, Other White, Mixed, Asian, Black, and Other.

GHQ questions include: "Have you recently been feeling unhappy or depressed?" and "Have you recently felt constantly under strain?", with response options including "Not at all", "No more than usual", "Rather more than usual" and "Much more than usual".

Table S1: Sample size and descriptive statistics for each Understanding Society wave and the pooled analysis.

	2010-11	2011-13	2012-14	2013-15	2014-16	2015-17	2016-18	2017-19	Pooled
Sample (N)	4404	4191	3944	3902	4283	3854	3638	3277	11,519
Higher Education group (%)	42.1	42.5	44.2	47.4	48.5	48.1	48.1	48.1	43.9
Female (%)	55.5	54.9	53.0	52.9	52.7	52.8	53.3	54.0	52.4
Mean age	20.1	20.1	20.0	20.1	20.2	20.3	20.3	20.3	19.8
White British (%)	77.3	75.4	75.4	75.2	65.7	66.8	68.1	69.2	68.5
Highest parental educational qualification (%)									
Degree or higher	35.3	39.3	41.1	43.4	43.2	44.4	45.7	48.2	40.6
A level or eqv.	22.3	21.3	21.0	20.2	19.2	19.7	19.5	18.4	20.4
GCSE or eqv.	23.9	21.4	20.9	20.1	20.9	19.8	19.6	20.6	21.6
No qual.	11.6	11.5	10.1	9.3	9.7	9.0	8.4	6.4	10.4
Other	6.9	6.6	6.9	7.1	6.9	7.1	6.8	6.4	6.9

Table S2: Sample size and descriptive statistics for higher education (HE) and non-higher education (non-HE) groups. Results are weighted means and percentages. p-Values are from chi square tests and t-tests.

	2010-2011			2011-2013			2012-2014			2013-2015		
	HE	Non-HE	p-value	HE	Non-HE	p-value	HE	Non-HE	p-value	Student	Non-student	p-value
Sample (N)	1,852	2,552	-	1,780	2,411	-	1,745	2,199	-	1,848	2,054	-
Mean age	20.3	19.9	<0.001	20.4	19.8	<0.001	20.4	19.7	<0.001	20.4	19.8	<0.001
Female (%)	56.0	55.1	-	55.5	54.5	-	55.1	51.3	<0.05	53.3	52.5	-
White Brit. (%)	71.2	81.7	<0.001	71.8	78.1	<0.001	71.7	78.4	<0.001	71.4	78.6	<0.001
Highest parental educational qualification (%)												
Degree or higher	43.0	29.7		48.1	32.8		49	34.8		50.9	36.7	
A level or eqv.	21.4	23.0		20.1	22.2		19.0	22.6		18.3	21.9	
GCSE or eqv.	19.5	27.2	<0.001	17.2	24.4	<0.001	18.0	23.2	<0.001	17.4	22.5	<0.001
No qual.	10.3	12.6		9.5	12.8		7.9	11.9		7.0	11.3	
Other	5.8	7.6		5.0	7.8		6.1	7.6		6.4	7.7	
	**2014-16**			**2015-17**			**2016-18**			**2017-19**		
	HE	Non-HE	p-value	HE	Non-HE	p-value	HE	Non-HE	p-value	HE	Non-HE	p-value
Sample (N)	2,079	2,204	-	1,852	2,002	-	1,748	1,890	-	1,576	1,576	-
Mean age	20.6	19.8	<0.001	20.7	19.9	<0.001	20.7	19.9	<0.001	20.8	19,84	<0.001
Female (%)	54.1	51.4	-	54.7	51.1	<0.05	55.4	51.4	<0.05	54.4	53.5	-
White Brit. (%)	60.4	70.6	<0.001	62.0	71.2	<0.001	63.2	72.7	<0.001	64.6	73.4	<0.001
Highest parental educational qualification (%)												
Degree or higher	50.2	36.7		51.2	38.1		53.1	38.9		55.0	42.0	
A level or eqv.	18.6	19.8		18.9	20.4		18.7	20.4		17.2	19.6	
GCSE or eqv.	17.1	24.4	<0.001	23.0	23.0	<0.001	15.9	9.7	<0.001	16.8	24.1	<0.001
No qual.	8.1	11.3		10.2	10.2		6.9	7.9		5.8	6.9	
Other	6.0	7.9		5.7	8.3		5.5	7.9		5.3	7.4	

Table S3: Parameter estimates of higher education status on psychological distress (GHQ score).

	2010-11	2011-13	2012-14	2013-15	2014-16	2015-17	2016-18	2017-19	Pooled
	β (low 95% CI, high 95% CI)	β (low 95% CI, high 95% CI)	β (low 95% CI, high 95% CI)	β (low 95% CI, high 95% CI)	β (low 95% CI, high 95% CI)	β (low 95% CI, high 95% CI)	β (low 95% CI, high 95% CI)	β (low 95% CI, high 95% CI)	β (low 95% CI, high 95% CI)
HE vs non-HE (ref category)									
Unadjusted	-0.39 (-0.81, 0.04)	-0.09 (-0.54, 0.37)	0.24 (-0.28, 0.75)	-0.38 (-0.86, 0.10)	-0.15 (-0.69, 0.40)	-0.43 (-0.98, 0.13)	-0.14 (-0.78, 0.49)	-0.29 (-0.92, 0.36)	-0.36* (-0.65, -0.08)
Adjusted[a]	-0.52* (-0.94, -0.10)	-0.20 (-0.65, 0.25)	0.05 (-0.48, 0.57)	-0.35 (-0.84, 0.13)	-0.38 (-0.92, 0.16)	-0.58* (-1.11, -0.04)	-0.32 (-0.94, 0.30)	-0.32 (-0.97, 0.34)	-0.37* (-0.66, -0.08)
Male HE vs non-HE (ref category)									
Adjusted[a]	-0.31 (-0.90, 0.27)	0.15 (-0.45, 0.74)	0.08 (-0.63, 0.80)	-0.08 (-0.77, 0.62)	-0.27* (-1.00, 0.46)	-0.21 (-0.91, 0.49)	-0.02 (-0.87, 0.82)	-0.67 (-1.68, 0.33)	-0.27 (-0.65, 0.10)
Female HE vs non-HE (ref category)									
Adjusted[a]	-0.67* (-1.30, -0.05)	-0.54 (-1.26, 0.19)	0.02 (-0.71, 0.75)	-0.68 (-1.43, 0.07)	-0.53* (-1.42, 0.36)	-0.94* (-1.73, -0.15)	-0.62 (-1.48, 0.23)	0.19 (-0.78, 1.15)	-0.44 (-0.89, 0.01

a. adjusted for age, sex, ethnicity and parental education in all models and additionally for study year in the pooled analysis.

The non-higher education group is the reference category (* indicates p-value is <0.05)

Table S4: Odds ratios of higher education status on psychological distress caseness

	**2010-11**	**2011-13**	**2012-14**	**2013-15**	**2014-16**	**2015-17**	**2016-18**	**2017-19**	**Pooled**
	Odds ratio (low 95% CI, high 95% CI)	Odds ratio (low 95% CI, high 95% CI)	Odds ratio (low 95% CI, high 95% CI)	Odds ratio (low 95% CI, high 95% CI)	Odds ratio (low 95% CI, high 95% CI)	Odds ratio (low 95% CI, high 95% CI)	Odds ratio (low 95% CI, high 95% CI)	Odds ratio (low 95% CI, high 95% CI)	Odds ratio (low 95% CI, high 95% CI)
HE vs non-HE (ref category)									
Unadjusted	0.92 (0.76, 1.11)	0.92 (0.78, 1.09)	1.10 (0.92, 1.32)	0.83* (0.69, 1.00)	0.93* (0.76, 1.14)	0.91* (0.74, 1.13)	0.99 (0.81, 1.22)	0.88 (0.70, 1.11)	0.91 (0.81, 1.02)
Adjusted[a]	0.88 (0.73, 1.07)	0.89 (0.75, 1.06)	1.04 (0.86, 1.25)	0.83* (0.69, 1.00)	0.87 (0.69, 1.08)	0.86 (0.69, 1.07)	0.90 (0.73, 1.12)	0.84 (0.66, 1.07)	0.91 (0.81, 1.02)
Male HE vs non-HE (ref category)									
Adjusted[a.]	0.89 (0.65, 1.23)	1.01 (0.77, 1.32)	1.15 (0.85, 1.55)	0.82 (0.61, 1.09)	0.89 (0.62, 1.27)	0.98 (0.68, 1.39)	1.00 (0.74, 1.41)	0.76 (0.51, 1.13)	0.92 (0.77, 1.09)
Female HE vs non-HE (ref category)									
Adjusted[a.]	0.88 (0.68, 1.14)	0.80 (0.62, 1.04)	0.95 (0.73, 1.23)	0.83 (0.64, 1.08)	0.83 (0.63, 1.11)	0.76 (0.57, 1.02)	0.82 (0.62, 1.08)	1.00 (0.72, 1.38)	0.90 (0.77, 1.06)

a. adjusted for age, sex, ethnicity and parental education in all models and additionally for study year in the pooled analysis.

The non-higher education group is the reference category (* indicates p-value is <0.05.)

Table S5: Parameter estimates of higher education status on psychological distress (GHQ score) – sensitivity analyses.

	**Pooled**
	β (low 95% CI, high 95% CI)
Excluding completed graduates HE vs non-HE (ref category) N=10,489	
Unadjusted	-0.51* (-0.82, 0.20)
Adjusted[a]	-0.44* (-0.76, -0.13)
Age 17-21 HE vs non-HE (ref category) N=7,637	
Unadjusted	-0.37* (-0.71, 0.04)
Adjusted[a]	-0.30 (-0.64, 0.04)

a. adjusted for age, sex, ethnicity and parental education in all models and additionally for study year in the pooled analysis.
The non-higher education group is the reference category (* indicates p-value is <0.05)
